# Diagnosis of congenital and acquired Gerbode defects by cardiac MRI: two cases

**DOI:** 10.1016/j.radcr.2025.11.027

**Published:** 2025-11-29

**Authors:** Quang Trung Nguyen, Viet Dung Le, Thi Minh Ly Nguyen, Ngoc Cuong Nguyen, Tuan Linh Le

**Affiliations:** a Radiology Center, Hanoi Medical University Hospital, Hanoi, Vietnam; b Cardiology Center, Hanoi Medical University Hospital, Hanoi, Vietnam; c Department of Radiology, Hanoi Medical University, Hanoi, Vietnam; d Department of Cardiology, Hanoi Medical University, Hanoi, Vietnam

**Keywords:** Gerbode defect, Cardiac magnetic resonance, Left-To-Right shunt, Ventricular septal defect, Transesophageal echocardiography

## Abstract

The Gerbode defect is a rare left ventricle-to-right atrium shunt, often misdiagnosed as tricuspid regurgitation, and may be either congenital or acquired. We report two cases: one acquired Gerbode defect following redo mechanical mitral valve replacement surgery, presenting with dyspnea and progressive heart failure; and one incidental congenital Gerbode defect in an asymptomatic patient. In both cases, Cardiac Magnetic Resonance (CMR) clarified shunt anatomy and informed management decisions. The first patient underwent successful percutaneous closure under Digital Subtraction Angiography (DSA), while the second received conservative follow-up due to the absence of symptoms. CMR complemented echocardiography by delineating anatomical features, particularly when acoustic windows were limited, and supported post-treatment evaluation.

## Introduction

The Gerbode defect is a rare abnormal communication between the left ventricle (LV) and the right atrium (RA), representing a rare form of congenital heart disease [1] of all congenital heart diseases, resulting in an unusual left-to-right intracardiac shunt. Besides congenital causes, acquired Gerbode defects can occur in the context of infective endocarditis, postcardiac surgery, or blunt chest trauma.

Due to the anatomical location of the shunt near the atrioventricular septum, Doppler echocardiography may misinterpret it as tricuspid regurgitation or pulmonary hypertension-especially in inexperienced echocardiographers unfamiliar with Gerbode-type defects. In acquired cases, echocardiographic evaluation becomes more challenging due to the presence of prosthetic valves or complex septal lesions, which may obscure clear visualization.

Hence, multimodality imaging—particularly CMR—plays a crucial role in accurately diagnosing the lesion location, assessing flow characteristics, and evaluating anatomical features and providing volumetric surrogates of hemodynamic status, particularly when direct flow measurements are unavailable.

In this article, we present two characteristic cases of Gerbode defect: one acquired postmitral valve replacement, and one incidental congenital case without symptoms, to highlight the diagnostic, treatment planning, and follow-up role of CMR, even in the absence of advanced sequences such as Phase-contrast or 4D Flow.

## Case presentation

Case 1: A 47-year-old female presented with progressively worsening exertional dyspnea over a 2-week period. She denied chest pain or fever. Her past medical history was notable for a redo mechanical mitral valve replacement three months earlier, performed due to degeneration of a previous bioprosthetic valve. She had no history of hypertension or diabetes and was maintained on oral vitamin K antagonist therapy.

On physical examination, her blood pressure was 110/70 mmHg and heart rate was 85 bpm. A mechanical valve click was audible, along with a grade 3/6 systolic murmur along the left sternal border. Clinical signs of right heart failure were evident, including jugular venous distension, hepatomegaly, and mild bilateral lower limb edema.

**Fig. 1:**
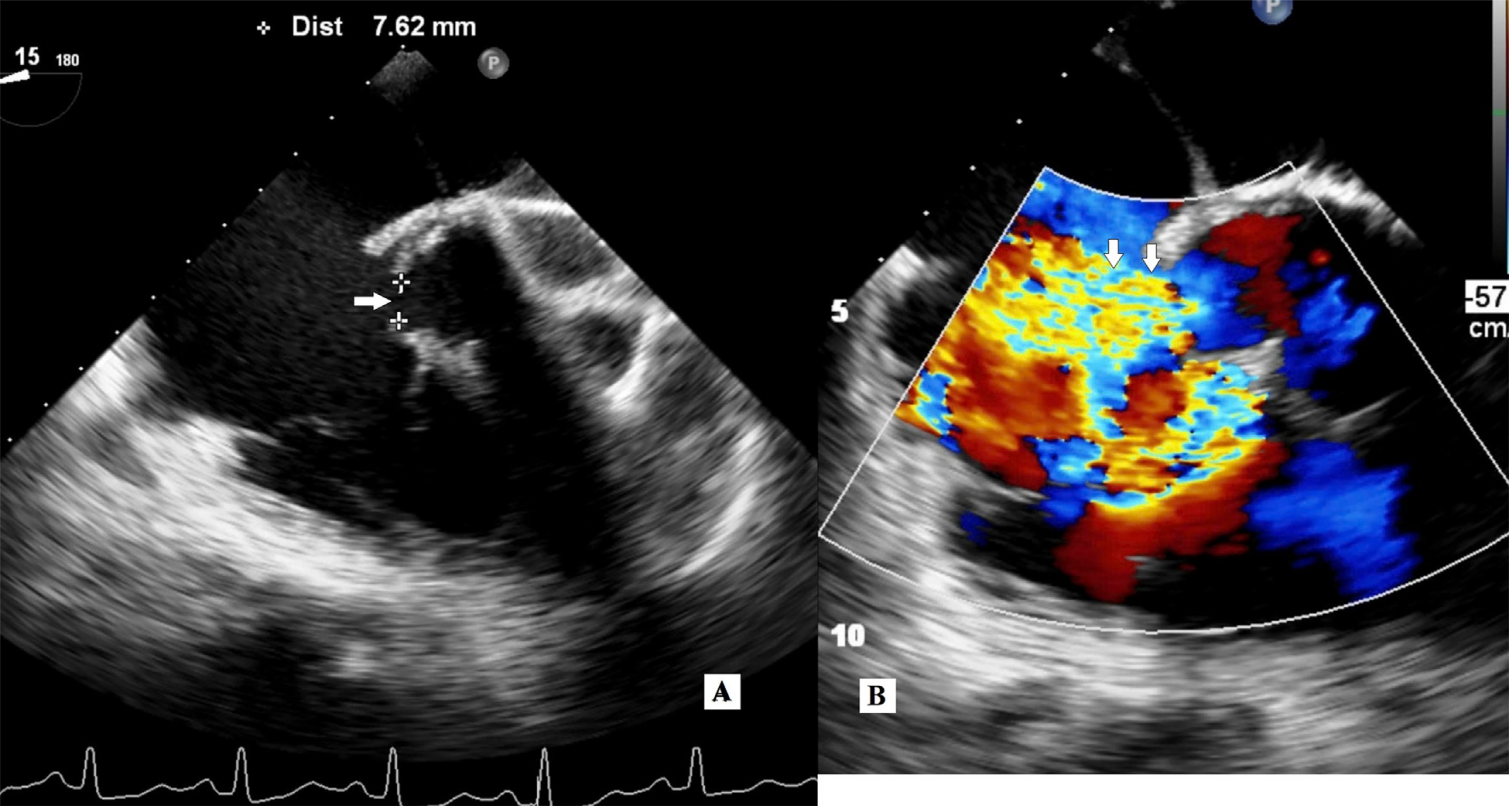
TEE in the first patient. (A) Mid-esophageal long-axis 2D view (∼0–15°) shows a rupture in the membranous portion of the atrioventricular septum, forming a direct LV-RA shunt. The defect measures 7.6 mm (calipers) and is located superior to the tricuspid valve septal leaflet (white arrow). (B) Color Doppler in the same view demonstrates a high-velocity left-to-right jet with turbulent flow entering the right atrium posterior to the tricuspid valve, consistent with a direct (supravalvular) Gerbode-type defect (white arrow). Velocity scale: ±57.4 cm/s.

**Fig. 2:**
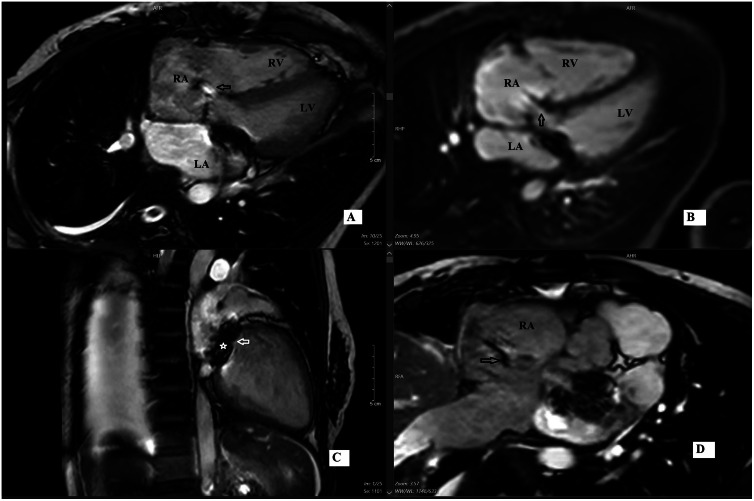
CMR imaging using SSFP sequences in various views demonstrates an abnormal shunt from the left ventricle (LV) to the right atrium (RA) through the atrioventricular membranous septum (A) Cine 4-chamber view shows a direct LV-RA shunt (black arrow). (B) First-pass perfusion in a 4-chamber view, acquired between standard short-axis slices, visualizes the shunt ascending from the lower interventricular septum into the RA (black arrow). (C) Cine 2-chamber long-axis view showing the mechanical mitral valve (*–white arrow). (D) Cine short-axis basal view showing mild RA dilation and LV-RA shunt (black arrow). RA, right atrium; LA, left atrium; RV, right ventricle; LV, left ventricle.

Transesophageal echocardiography (TEE) revealed a high-velocity left ventricular to right atrial (LV-RA) shunt measuring approximately 7 mm (Fig. 1), originating from the left ventricle (LV) and entering the (RA). The jet was directed anteriorly, toward the RA side, and coursed just above the septal leaflet of the tricuspid valve. This directional pattern confirmed a Gerbode-type defect rather than tricuspid regurgitation. CMR, performed on a 1.5T system using cine balanced steady-state free precession (bSSFP) sequences in long-axis and short-axis views, confirmed the presence of a direct LV-RA shunt through the atrioventricular membranous septum (Fig. 2). Quantitative functional assessment demonstrated preserved left ventricular function with an ejection fraction (LVEF) of 68%, end-diastolic volume (EDV) of 183 mL, end-systolic volume (ESV) of 58 mL, and indexed end-diastolic volume (EDV/BSA) of 131 mL/m². The stroke volume indexed to body surface area was 90 mL/m², and the calculated cardiac output was 10.6 L/min. The left ventricular myocardial mass was 111 g.

Right ventricular (RV) evaluation showed an ejection fraction (RVEF) of 51%, end-diastolic volume (EDV) of 258 ml, end-systolic volume (ESV) of 125 mL, and indexed EDV (EDV/BSA) of 185 mL/m², consistent with mild right ventricular dilation. No significant right atrial enlargement was observed based on visual assessment, and the main pulmonary artery measured 35 mm in diameter, while the ascending aorta measured 27 mm

The defect measured 7.6 mm in diameter (inner-to-inner edge at the defect neck), with normal left and right ventricular size and function, no additional septal defects, and no significant valvular regurgitation apart from the shunt jet. The left ventricular ejection fraction (LVEF) was preserved at 68%.

Under the guidance of DSA, the patient underwent percutaneous closure of the ventricular septal defect via femoral arterial and venous access, as well as right radial artery access.

During the initial procedure, an 18 × 20 mm ventricular septal defect (VSD) occluder was deployed. However, postprocedural transthoracic echocardiography (TTE) and CMR revealed a residual shunt measuring 2.8-3.0 mm.

**Fig. 3:**
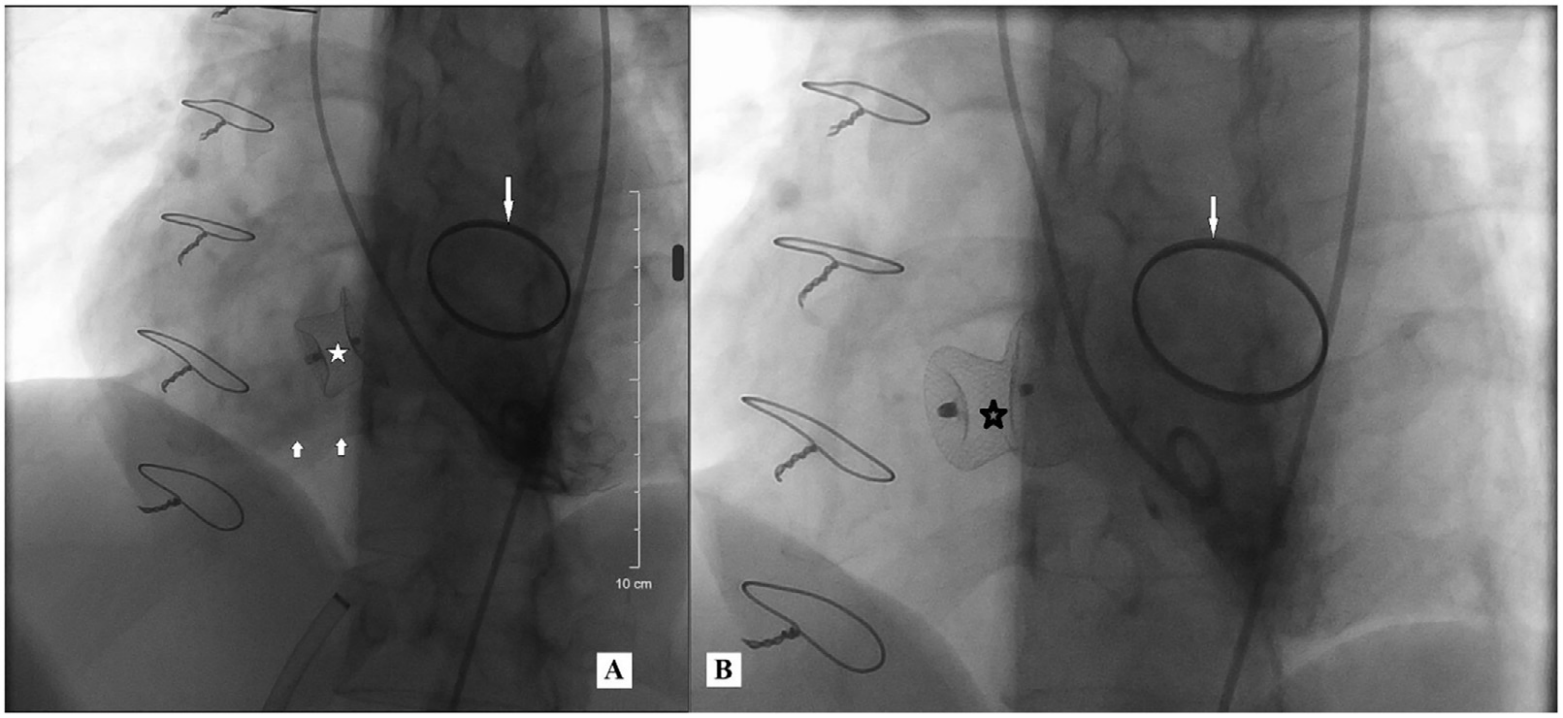
Digital subtraction angiography (DSA) during transcatheter closure of the Gerbode defect in the first patient. (A) Injection from the left ventricle after deployment of an 18 × 20 mm occluder device *(*, white star) reveals a small residual shunt across the device (short white arrow). (B) Following replacement with a 20 × 22 mm occluder (, black star), no residual flow is seen. The mechanical mitral valve ring is also visible (long white arrow). Arrow key: short white = residual jet; long white = valve ring; star = device.

A second procedure was subsequently performed, during which the device was exchanged for a larger VSD occluder (LifeTech 20 × 22 mm) due to incomplete sealing caused by under-sizing of the original device. The total fluoroscopy time for the second procedure was 21 minutes and 50 seconds. Final angiographic assessment confirmed accurate device positioning with complete closure and no residual flow (Fig. 3).

**Fig. 4:**
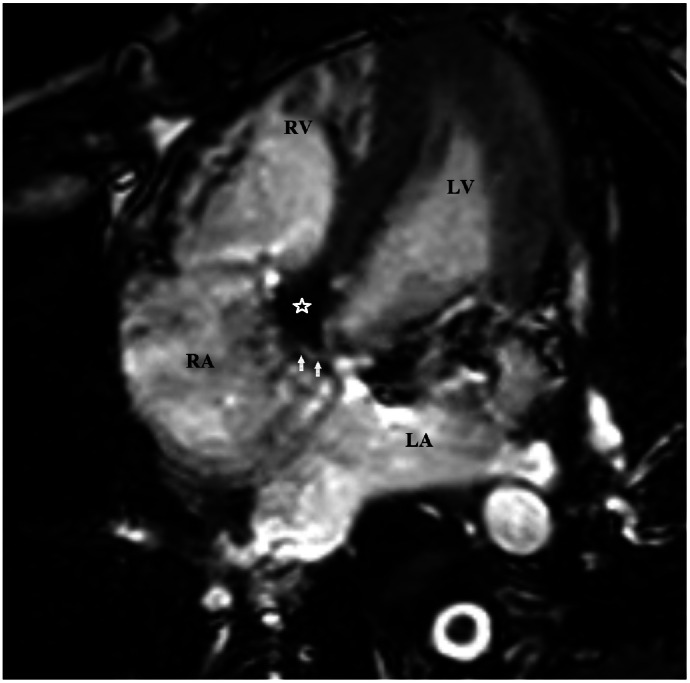
Cine MRI in a 4-chamber view after the first transcatheter closure in the first patient. residual left-to-right shunt (white arrow) is still visible alongside the implanted LifeTech 20 × 22 mm occluder device (*). This residual jet was eliminated after a second procedure.

Follow-up: Postprocedure transthoracic echocardiography and CMR showed a small residual shunt of 2.8-3.0 mm after the first intervention (Fig. 4). Following the second procedure, no residual shunt was observed. LVEF remained at 68%, and the patient was symptom-free and classified as NYHA functional class I during follow-up. The follow-up plan included TTE at 1, 3, and 6 months. CMR was scheduled at 6 months if symptoms reappeared or abnormalities were suspected.

Case 2: A 56-year-old woman was referred for further evaluation after a systolic murmur was incidentally detected during a routine health check-up. She reported no symptoms such as dyspnea, chest pain, or fatigue. Her past medical history was significant for chronic hypertension for over 10 years, well-controlled with a combination of an angiotensin-converting enzyme (ACE) inhibitor and a diuretic. She had no prior cardiac surgery or intervention, was a nonsmoker, did not consume alcohol, and had a family history of ischemic heart disease.

On clinical examination, her blood pressure was 130/80 mmHg, and heart rate was 78 bpm. A soft systolic murmur was auscultated along the left sternal border. No peripheral signs of heart failure were present, and physical examination was otherwise unremarkable.

**Fig. 5:**
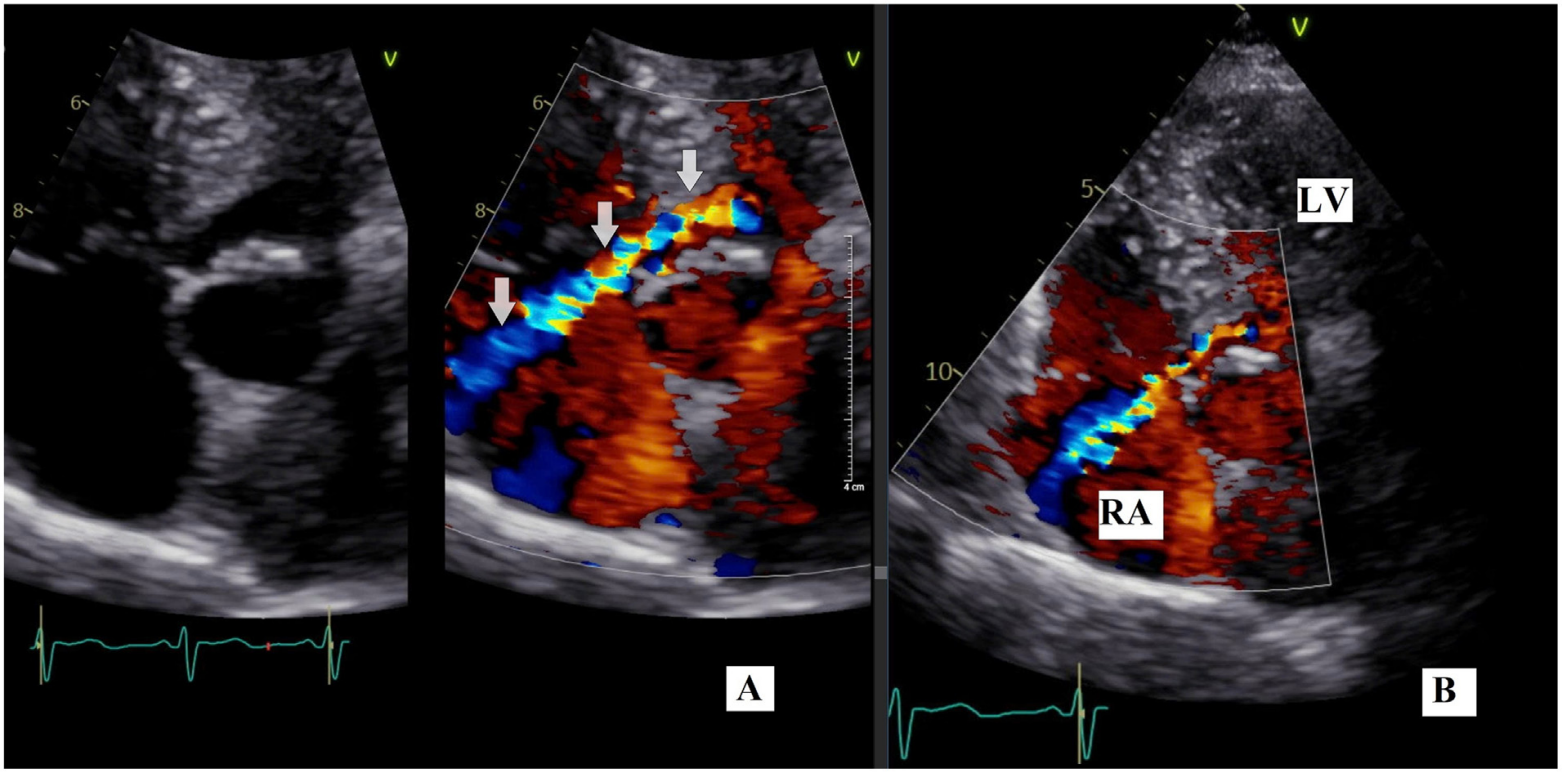
TTE in the second patient. (A-B) 2D and color Doppler views demonstrate a small left-to-right shunt from the left ventricle to the right atrium (white arrow).

**Fig. 6:**
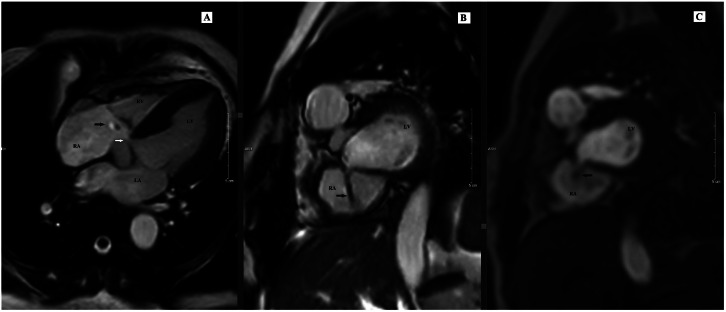
CMR in the second patient. CMR using steady-state free precession (SSFP) sequences in multiple views demonstrates a left-to-right shunt through the atrioventricular membranous septum (A) Cine 4-chamber view shows the defect (white arrow) and direct shunt jet from the left ventricle (LV) to the right atrium (RA) (black arrow). (B) Cine short-axis basal view illustrates the ascending jet direction toward the RA (black arrow). (C) First-pass perfusion in short-axis view confirms early contrast opacification of the RA from the shunt. Arrow key: white = defect neck; black = shunt flow.

TTE revealed a left ventricle-to-right atrium (LV-RA) shunt measuring approximately 4 mm, with a continuous left-to-right flow pattern. Continuous-wave Doppler recorded a peak gradient of 150 mmHg across the defect, consistent with a high-velocity jet. No tricuspid regurgitation was observed, and right ventricular function remained normal. No evidence of tricuspid regurgitation was observed, and right ventricular size and function were within normal limits (Fig. 5). CMR confirmed the presence of a small congenital Gerbode defect. There was no associated hemodynamic compromise or structural cardiac abnormality, and biventricular function was preserved (Fig. 6). Quantitative analysis revealed preserved left ventricular function, with a left ventricular ejection fraction (LVEF) of 62%, end-diastolic volume (EDV) of 151 mL, end-systolic volume (ESV) of 57 mL, and an indexed EDV (EDV/BSA) of 113 mL/m². The stroke volume was 94 mL, with a cardiac output of 7.5 L/min. The left ventricular mass was approximately 93 g.

Right ventricular assessment showed an ejection fraction (RVEF) of 53%, EDV of 95 mL, ESV of 45 mL, and a stroke volume of 50 mL, with a calculated cardiac output of 4.0 L/min. The ascending aorta and main pulmonary artery diameters were within normal limits, and no chamber enlargement was observed. Given the absence of clinical symptoms and the small size of the shunt, a conservative management approach was adopted. This decision was supported by normal right ventricular (RV) function, no right atrial (RA) enlargement, and no evidence of pulmonary hypertension on imaging. The patient was scheduled for regular follow-up, including TTE at 6-month intervals to monitor defect size and RV function. Cardiac magnetic resonance (CMR) was reserved for instances of clinical progression or inconclusive echocardiographic findings. The patient remained asymptomatic during follow-up. The clinical and imaging characteristics of both patients are compared in (Table 1).

**Table 1:** Comparative summary of clinical characteristics, imaging findings, and management of the two Gerbode defect cases.

Characteristic	Case 1: Acquired Gerbode defect postsurgery, symptomatic	Case 2: Congenital Gerbode defect, asymptomatic
Age / Gender	47 y old, female	56 y old, female
Medical history	Redo mechanical mitral valve replacement due to degenerated bioprosthetic valve	Chronic hypertension
Reason for detection	Dyspnea, progressive heart failure	Systolic murmur detected during routine check-up
Shunt size	∼7.6 mm	∼4 mm
Echocardiography	TEE suggested LV-RA shunt	TTE detected a small shunt
CMR findings	Clearly defined direct shunt and lesion location	Confirmed small Gerbode defect with no hemodynamic impact
Classification type	Direct (supravalvular)	Direct (supravalvular)
RV/RA indices	Indexed RV EDV: 185 mL/m²; RA enlarged	Normal RV volume and function; No RA enlargement
Management approach	Catheter-based intervention with DSA guidance; occlusion using LifeTech device	Conservative management with regular monitoring
Follow-up outcome	No residual shunt; symptom-free and stable after 3 months	Stable, no progression

## Discussion

### Pathogenesis and classification of the Gerbode defect

The Gerbode defect is a rare abnormal communication between the left ventricle (LV) and the (RA), creating a direct left-to-right shunt due to the high-pressure gradient between these chambers. Although originally described as a rare congenital anomaly, the incidence of acquired forms has been increasing in modern clinical practice due to the growing number of cardiovascular procedures [2].

From an embryological and pathological anatomy standpoint, the Gerbode defect arises from a lesion in the membranous portion of the atrioventricular septum, an area normally separated by the septal leaflet of the tricuspid valve. In this condition, blood from the LV can pass through a defect in the membranous septum and flow directly into the RA [2].

The defect is classified into three main types based on its anatomical relationship to the tricuspid valve [2]. The supravalvular type (also referred to as the direct type) is characterized by a shunt located above the septal leaflet of the tricuspid valve and accounts for approximately 32%-76% of reported cases, depending on the study population. The infravalvular type (or indirect type) involves a left-to-right shunt from the (LV) into the right ventricle (RV), followed by regurgitant flow through the tricuspid valve into the (RA); this form is frequently misdiagnosed as isolated tricuspid regurgitation. The mixed type**,** also known as the combined or valvular form, represents a combination of both the supravalvular and infravalvular patterns and is less commonly encountered.

A systematic analysis of 234 patients by S.-M. Yuan [3] found that most acquired cases were iatrogenic (51.1%), usually following cardiac surgery, particularly mitral or aortic valve replacement. Other causes included infection (36.7%), blunt chest trauma (9.3%), and myocardial infarction (3%). The first case in our study is a representative example of an acquired Gerbode defect following redo mechanical mitral valve replacement. This defect corresponds to a direct (supravalvular) Gerbode defect, as the shunt originates above the septal leaflet of the tricuspid valve and flows directly from the LV to the RA

Conversely, congenital Gerbode defects are typically diagnosed in infancy or childhood but can also be incidentally detected in asymptomatic adults. The second case in our series is a classic illustration of this form. This case also represents a direct (supravalvular) variant, with flow across the membranous septum entering the RA superior to the tricuspid septal leaflet. Winter et al. [4] reviewed 78 congenital case reports and found a wide range of clinical presentations, from asymptomatic murmurs to overt symptoms such as developmental delay, dyspnea, palpitations, and fatigue .

The clinical impact of the shunt depends on the size and anatomical location of the defect. Larger shunts may cause right atrial dilation, pulmonary hypertension, and right heart failure. Al-Sarraf et al. [5] described a congenital Gerbode defect measuring up to 15 mm that led to significant right heart failure, highlighting the hemodynamic disparity compared to smaller lesions.

### Role of CMR in diagnosis and management

In both clinical cases presented, CMR proved to be highly effective in identifying the exact location of the shunt, the direction of blood flow, and its relationship to valvular structures. This is particularly critical in postsurgical or postintervention patients, where echocardiographic windows may be compromised by prosthetic material or scar tissue, thus limiting image quality and diagnostic accuracy [2,5].

In the first patient, while TEE suggested a left-to-right shunt, only CMR clearly identified the direct LV-RA communication, localized the membranous septal rupture, and excluded other structural abnormalities. On echocardiography, a high-velocity jet was seen entering the (RA) above the tricuspid annulus, near the coronary sinus and posterior to the aortic root. Although this raised suspicion for tricuspid septal or posterior leaflet pathology, CMR clarified the anatomy, confirming a direct LV-RA shunt and excluding valvular involvement. This comprehensive assessment ruled out alternative causes of pressure gradient and supported the decision for device-based percutaneous intervention. Follow-up CMR was subsequently employed to evaluate residual shunting and intervention outcomes [5,6].

In the second case, where the patient was asymptomatic, CMR confirmed the presence of a small congenital Gerbode defect and excluded any significant hemodynamic consequences. This enabled the cardiology team to pursue a conservative management strategy, avoiding unnecessary procedures [4].

Beyond providing high-resolution anatomical details, CMR also offers hemodynamic quantification through advanced sequences such as Phase-contrast MRI and 4D Flow, which are especially useful in well-equipped medical centers. Although these techniques were not applied in our two cases, their potential has been widely validated and should be considered in complex Gerbode presentations or when precise Qp:Qs assessment is required to guide optimal clinical management [7,8,9].

Although advanced flow quantification techniques such as Phase-contrast MRI or 4D Flow MRI were not applied in our cases, their clinical utility is increasingly recognized. Prior studies have demonstrated that 4D Flow-derived Qp:Qs ratios closely correlate with invasive catheterization measurements, offering a noninvasive alternative for assessing shunt severity [9]. Furthermore, recent literature supports the expanding role of 4D Flow MRI in the long-term follow-up of adults with congenital heart disease [10]. While limited by availability in some regions, these advanced techniques may complement standard CMR protocols and should be considered when precise hemodynamic data are necessary.

### Comparison with previous studies

Several prior studies have documented the role of CMR in diagnosing Gerbode defects, particularly in postoperative cardiac patients. In the report by Cheema et al. [5] CMR enabled precise localization of the direct LV–RA shunt and supported the decision for endovascular intervention. Similarly, Chaturvedi et al.[6] described a case postaortic valve replacement in which CMR provided crucial supplementary information to clarify the nature of a suspected shunt not definitively visualized by echocardiography.

Our first clinical case mirrors these scenarios, where echocardiography suggested an abnormality, but only CMR confirmed the precise anatomical characteristics of the defect. A notable distinction in our study is that CMR was employed not only for diagnosis but also for postintervention monitoring**,** a facet not widely addressed in the current literature.

For the second case—an asymptomatic patient—the clinical scenario closely aligns with congenital Gerbode defects described by Winter et al. [4]. However, the application of CMR to confirm minimal hemodynamic impact and to delineate anatomical features helped guide a more informed choice for conservative management. This highlights the potential of CMR in risk stratification and long-term surveillance strategies.

Beyond anatomical delineation, recent studies have expanded the utility of CMR through advanced sequences like 4D Flow MRI**.** Notably, Zamani-Aliabadi et al. [7] demonstrated that Qp:Qs ratios derived from 4D Flow MRI were comparable to those obtained by cardiac catheterization (r = 0.962) in pediatric patients with ventricular septal defects. While this technique was not used in our cases, such evidence supports its promising role in the noninvasive evaluation of complex Gerbode defects**.**

Compared to previous reports, our study not only reaffirms the diagnostic value of CMR in Gerbode defects but also underscores its potential in postintervention and postoperative assessment, an area less emphasized in existing literature. The first case in particular illustrates a proactive imaging-based strategy that may enhance clinical management of acquired postoperative defects.

## Conclusion

CMR played a decisive role in clarifying diagnosis and guiding management strategies in both cases. In the acquired Gerbode defect, CMR enabled precise anatomical localization and supported device-based intervention planning. In the congenital case, CMR confirmed preserved cardiac function and anatomical integrity, justifying a conservative follow-up approach.

## Limitations and future directions

The primary limitation of this report is the small sample size and the lack of precise hemodynamic quantification. However, volumetric surrogates—particularly right ventricular size and function—were used to assess hemodynamic burden indirectly. Advanced flow imaging modalities, such as Phase-contrast or 4D Flow MRI, were not performed due to equipment and resource limitations at the imaging center during the study period. Additionally, the application of CMR faces several practical challenges, including contraindications in patients with magnetic implants (eg, pacemakers, metallic clips) or claustrophobia. The prolonged scan time and the need for patient stillness can also present difficulties, especially in uncooperative individuals or those with poor breath control—commonly encountered in elderly patients or young children. These factors must be considered carefully in clinical settings.

In Vietnam, 4D Flow MRI remains relatively uncommon in healthcare facilities due to limitations in equipment availability, image processing software, and specialized personnel training. However, as advanced MRI centers continue to emerge and the demand for noninvasive hemodynamic assessment grows, 4D Flow MRI could become a powerful tool for evaluating congenital heart anomalies such as the Gerbode defect.

Nonetheless, this study demonstrates the practical potential of standard CMR protocols even in settings where advanced flow sequences like 4D Flow are not yet available. Future research should explore direct comparisons between CMR and advanced imaging techniques such as 4D Flow MRI or cardiac CT, potentially guiding further development in diagnostic pathways and noninvasive management strategies.

## Patient consent

Informed consent for patient information to be published in this article was obtained.
